# A Matter of Life or Death: Productively Infected and Bystander CD4 T Cells in Early HIV Infection

**DOI:** 10.3389/fimmu.2020.626431

**Published:** 2021-02-12

**Authors:** Dechao Cao, Sushant Khanal, Ling Wang, Zhengke Li, Juan Zhao, Lam Nhat Nguyen, Lam Ngoc Thao Nguyen, Xindi Dang, Madison Schank, Bal Krishna Chand Thakuri, Jinyu Zhang, Zeyuan Lu, Xiao Y. Wu, Zheng D. Morrison, Mohamed El Gazzar, Shunbin Ning, Jonathan P. Moorman, Zhi Q. Yao

**Affiliations:** ^1^ Center of Excellence for Inflammation, Infectious Disease and Immunity, James H. Quillen College of Medicine, East Tennessee State University, Johnson City, TN, United States; ^2^ Division of Infectious, Inflammatory and Immunologic Diseases, Department of Internal Medicine, Quillen College of Medicine, East Tennessee State University, Johnson City, TN, United States; ^3^ Hepatitis (HCV/HBV/HIV) Program, James H. Quillen VA Medical Center, Department of Veterans Affairs, Johnson City, TN, United States

**Keywords:** AKT, ATM, HIV, telomerase, telomere, T cell death, survival

## Abstract

CD4 T cell death or survival following initial HIV infection is crucial for the development of viral reservoirs and latent infection, making its evaluation critical in devising strategies for HIV cure. Here we infected primary CD4 T cells with a wild-type HIV-1 and investigated the death and survival mechanisms in productively infected and bystander cells during early HIV infection. We found that HIV-infected cells exhibited increased programmed cell death, such as apoptosis, pyroptosis, and ferroptosis, than uninfected cells. However, productively infected (p24^+^) cells and bystander (p24^-^) cells displayed different patterns of cell death due to differential expression of pro-/anti-apoptotic proteins and signaling molecules. Cell death was triggered by an aberrant DNA damage response (DDR), as evidenced by increases in γH2AX levels, which inversely correlated with telomere length and telomerase levels during HIV infection. Mechanistically, HIV-infected cells exhibited a gradual shortening of telomeres following infection. Notably, p24^+^ cells had longer telomeres compared to p24^-^ cells, and telomere length positively correlated with the telomerase, pAKT, and pATM expressions in HIV-infected CD4 T cells. Importantly, blockade of viral entry attenuated the HIV-induced inhibition of telomerase, pAKT, and pATM as well as the associated telomere erosion and cell death. Moreover, ATM inhibition promoted survival of HIV-infected CD4 T cells, especially p24^+^ cells, and rescued telomerase and AKT activities by inhibiting cell activation, HIV infection, and DDR. These results indicate that productively infected and bystander CD4 T cells employ different mechanisms for their survival and death, suggesting a possible pro-survival, pro-reservoir mechanism during early HIV infection.

## Introduction

HIV infection induces a gradual depletion of CD4 T cells, leading to decline of host immunity which increases the risk of opportunistic infections and elevates mortality (1, 2). HIV infection, if left untreated, can lead to AIDS over a period of 8-10 years. This disease progression was originally thought to be a result of slow but inexorable virus-mediated CD4 T cell destruction. However, recent studies have shown that massive CD4 T cell depletion occurs quite early in HIV-1 infection (1, 2). In most HIV-infected individuals, this initial phase of destruction is counteracted by CD4 T cell regeneration and compensatory responses. However, this T compensatory mechanism is short-lived, promotes immune activation and dysregulation, and cannot restore all functionally competent CD4 T cell populations. Ultimately, these processes disrupt CD4 T cell homeostasis and reduce the levels of critical effector/memory T cells below the threshold necessary to prevent opportunistic infections or associated pathologies.

Antiretroviral therapy (ART) suppresses HIV replication and enhances CD4 T cell recovery. However, complete CD4 T cell recovery or immune reconstitution does not always occur despite a successful ART-mediated control of HIV replication (3, 4). In addition, with ART treatment HIV develops latent infection with the existence of viral reservoirs, thus posing a constant risk of viral rebound in the absence of optimal ART. these HIV reservoirs represent a major barrier to HIV eradication and likely drive chronic inflammation and incomplete immune reconstitution (5, 6). As the challenges inherent to these viral reservoirs have come to be recognized over the last decade, it is clear that evaluations of the fundamental mechanisms facilitating HIV reservoir formation is critical to understanding HIV persistence.

HIV latency is established early in both resting and activated primary CD4 T cells, and HIV reservoir sizes and persistence are driven by T cell survival and homeostatic proliferation (7, 8). One possible mechanism underlying the development of these reservoirs is the preservation of the infected CD4 memory T cells that carry integrated provirus and survive cell death (5–9). While these viral reservoirs can be eliminated through virus-induced cytopathogenic effects or T cell receptor (TCR) activation-induced cell death, their reversion to a virus-silenced and/or a cell-resting state facilitates survival during HIV latency. Unknown machineries are likely to contribute to the survival of select HIV-infected cells, leading to the establishment of HIV reservoirs.

We have recently shown that chronic viral (HIV, HBV, HCV) infection can induce topological DNA damage and telomere erosion due to deficiency of topoisomerases (such as Top1 and Top2α), telomere shelterin protein (TRF2), and DNA repair kinases (such as ATM and ATR), all of which can lead to telomere shortening, genomic instability, and cell apoptosis (10–17). Productively infected T cells, however, can also affect bystander cells through secretion of inflammatory cytokines or induction of inhibitory molecules (18–20). Thus, immunodeficiency appears to be an overt result of CD4 T cell homeostasis in productively infected and bystander T cells, affecting their sensitivity to cell activation, proliferation, and vulnerability to cell death. This immunodeficiency mechanism is likely to be due to an interplay between direct viral cytopathogenicity and indirect inflammatory stimulation. Thus, investigating the fundamental mechanisms underlying CD4 T cell survival or death during early HIV infection will enhance our understanding of how HIV establishes its reservoirs, which is essential to devising strategies for eliminating HIV latency.

Using our established cellular model of *in vitro* HIV infection in primary CD4 T cells with or without ART (17), we investigated the mechanisms of telomere dynamics in CD4 T cell homeostasis and the role of TCR signaling pathways in cell death during early HIV infection. We analyzed the fate of productively infected (p24^+^) and bystander (p24^-^) CD4 T cells, the dynamics of their telomeres, and the state of TCR signaling pathways during early HIV infection. We discovered that HIV-infected cells exhibit increased programmed cell death, with gradual shortening of telomeres and inhibition of the TCR signaling pathways. Interestingly, productively infected CD4 T cells showed remarkably prolonged telomeres, increased levels of telomerase, and greater activation of TCR signaling pathways compared to bystander CD4 T cells, suggesting the involvement of cell survival and pro-reservoir mechanisms. These results indicate that telomere dynamics control T cell fate and that HIV-infected and uninfected cells behave differently in their survival and death mechanisms during early infection. Thus, disrupting these mechanisms may offer novel strategies to promote HIV-infected cell death so as to eliminate the virus reservoirs and rescue bystander cells to maintain the host immunity.

## Methods

### CD4 T Cell Isolation and HIV-1 Infection

Peripheral blood mononuclear cells (PBMCs) were isolated from whole blood of healthy subjects (HS; negative for HBV, HCV, and HIV infections) supplied by BioIVT (Gray, TN) using Ficoll density centrifugation (GE Healthcare; Piscataway, NJ). CD4 T cells were isolated from PBMCs using CD4 T Cell Negative Isolation Kit (Miltenyi Biotec, Auburn, CA). The CD4 T cells were cultured in complete RPMI-1640 medium containing 10% FBS (Atlanta Biologicals; Flowery Branch, GA), 100 IU/ml penicillin, and 2 mM L-glutamine (Thermo Scientific, Logan, Utah). The cells were stimulated with 1 µg/ml anti-CD3, 1 µg/ml anti-CD28 (BD Bioscience, San Jose, CA), 100 IU IL-2 (Sigma-Aldrich, St. Louis, MO) for 48 h. The cells were infected with HIV-1 as described previously (17). Briefly, 20 µg of pNL4-3 plasmid [contains full-length HIV-1 DNA inserted into a pUC18 vector) obtained from the NIH AIDS Reagent Program, originally deposited by Dr. Malcolm Martin (21)]. The plasmid DNA was transfected into the HEK293T cells using the polyethylenimine (PEI) method. The supernatants of HIV-transfected HEK293T cells were used to infect SupT-1 cells to prepare the virus-stock for CD4 T cell infection using the spinoculation method (21). Approximately 1 x 10^6^ CD4 T cells were infected with SupT-1 supernatant containing 1 x 10^6^ HIV-1 in culture plates using centrifugation at 1620 x g in a 37°C incubator. After 2 h of spinoculation, the supernatants were removed to discard the unattached viruses. Complete RPMI-1640 medium was added, and the cells were harvested at days 3, 5, and 7 for analysis. For blocking the virus entry or the ATM pathway, Maraviroc (2 µM) and T-20 (250 nM) (from the NIH AIDS Reagent Program), or ATM inhibitor (KU-60019, 10 μM) (Abcam, Cambridge, MA) were added to the culture after 2 h of inoculation. For HIV protein treatment, CD4 T cells were incubated with recombinant HIV proteins (gp120, p24, Tat, p7, Nef, and Gag) for 3, 5, and 7 days, followed by measuring telomere length, hTERT, pAKT, and pATM by flow cytometry.

### Flow Cytometry

For the apoptosis assay, cells were stained with PE Annexin V Apoptosis Detection Kit I (BD Bioscience, San Jose, CA). Briefly, the cells were harvested, washed with DPBS, labeled with Annexin V-PE and 7-AAD in binding buffer, and then analyzed by AccuriC6 Plus flow cytometer (BD Bioscience) and FlowJo software (Tree Star, Ashland, OR). For nuclear protein staining, the cells were fixed in Fixation Buffer (BioLegend, San Diego, CA), permeabilized with Fixation/Permeabilization Concentrate and Diluent (Thermo Fisher Scientific, Waltham, MA), and stained with Anti-HIV-1 p24 monoclonal antibody (KC57)-PE (NIH AIDS Reagent Program), γH2AX, PD-1 (BioLegend), pAKT, active caspase-3 kit (Thermo Fisher Scientific), active caspase-1 kit (Abcam, Cambridge, MA), hTERT (Rockland Immunochemicals, Limerick, PA), GPx-4, Bcl-2, Mcl-1, p-Bad, Bax, Ox40, Nur77, Birc5 and pATM (Santa Cruze Biotechnology, Dallas, TX). Isotype control antibodies (Thermo Fisher Scientific) and single staining controls were used to determine background staining and to adjust multicolor compensation as a gating strategy.

### Flow-FISH and Meta-FISH Analysis of Telomere Length

Flow-FISH analysis of telomere length was carried out as described previously (13–17). For meta-FISH analysis, CD4 T cells with or without HIV infection for 5 days were treated with 0.1 µg/ml colcemid for 2 h and harvested by centrifugation. The cells were then incubated in 75 mM KCl hypotonic buffer at 37°C for 10 or 20 min at room temperature, followed by fixation in methanol and glacial acid acetic (3:1). The cells were mounted on microscopic slides and dried overnight before fixation in 4% formaldehyde. The slides were treated with RNaseA and Pepsin at 37°C and then dehydrated in successive ethanol solutions of 70%, 90%, and 100%. Telomere hybridization was conducted using a FITC-488 conjugated telomeric PNA probe (PNA Bio, F1004 TelC-FITC488 (CCCTAA)3) at 37°C for 4 h, and the slides were washed in hybridization buffer and kept overnight at room temperature in Diamond AntiFade with DAPI (Invitrogen). Approximately 50 cells in meta-phase in each treatment were analyzed with a Leica SP8 confocal microscope, and the percentages (%) of fragile telomeres (signal of telomeres on chromosomes that break into segments) or telomere loss (without visible FISH signal) were calculated.

### Western Blot

Levels of γH2AX and hTERT proteins in CD4 T cells with or without HIV infection for 5 days were determined by immunoblotting. Primary and secondary antibodies included anti-γH2AX, hTERT and horseradish peroxide-conjugated antibody (Cell Signaling). Images were captured using ChemiDoc™ XRS+ System (Bio-Rad).

### Southern Blot

Southern blot was performed to determine telomere terminal restriction fragments (TRF), as previously described (22). Briefly, after cell treatment or HIV-infection, DNA was extracted and digested with *HinfI* and *RsaI* restriction enzymes to remove non-telemetric DNA. The DNA fragments were separated on 0.5% agarose gel, blotted, detected by a DIG-labeled (CCCTAA)_3_ probe, and visualized by chemiluminescence.

### Statistical Analysis

The data were analyzed using Prism 7 software and are presented as mean ± S.E. (standard error). The outliers were identified by the ROUT method (Q = 1.000%) and excluded from the analysis. Student’s t-test was used to compare means of two independent groups with equal variances, and Welch’s correction was utilized if unequal variances were found. Comparisons between two groups for skewed data were made using the nonparametric Mann-Whitney U test. The magnitude of correlation was appropriately measured with Pearson’s correlation coefficient (parametric approach) or Spearman’s correlation coefficient (nonparametric approach) based on the property of the datasets. *P*-values <0.05 or <0.01 were considered statistically significant or very significant, respectively.

## Results

### HIV Induces CD4 T Cell Death in Productively Infected and Bystander Cells During Early Infection *via* Different Mechanisms

**Figure 1:**
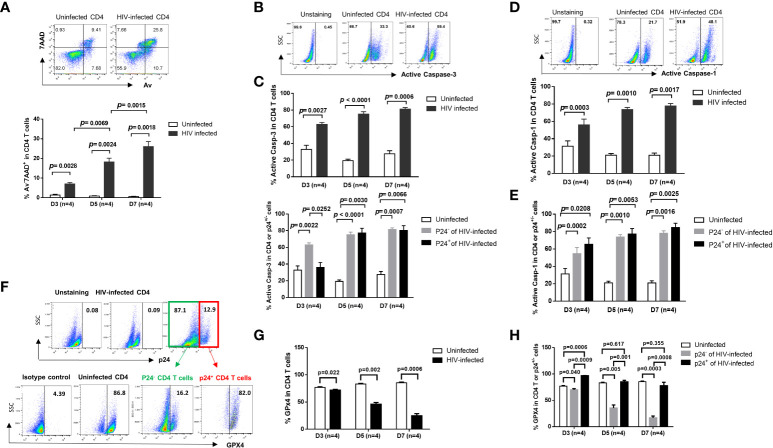
HIV induces CD4 T cell death in productively infected and bystander cells during early infection *via* different mechanisms. **(A)** Representative dot plots (day 7) and summary of the percentage (%) of Av and 7AAD levels in HIV-infected and uninfected CD4 T cells, as determined by flow cytometry. **(B, C)** Representative dot plots and summary data showing active caspase-3% in productively HIV-infected (p24^+^) and bystander (p24^-^) or uninfected cells at days 3, 5, and 7 in cultures. **(D, E)** Representative dot plots and summary data of active caspase-1% in HIV-infected (p24^+^) and bystander (p24^-^) or uninfected cells at day 3, day 5, and day 7. **(F–H)** Representative dot plots and summary data of GPX4% in HIV-infected (p24^+^) and bystander (p24^-^) or uninfected cells at days 3, 5, and 7.

Progressive depletion of CD4 T cells is a hallmark of untreated HIV infection (1, 2). To understand the mechanisms of CD4 T cell demise during HIV infection, we established an *in vitro* HIV-infected primary CD4 T cell culture system using a wild-type, replicable HIV NL4-3 strain (23). We have recently shown that HIV can productively infect human CD4 T cells, causing a DNA damage response (DDR) and programmed cell death in a time- and dose-dependent manner (17). Here, we further investigated the pathways of HIV-induced CD4 T cell death, including apoptosis, pyroptosis, and ferroptosis within this model. As shown in **Figure 1A**, HIV infection significantly increased cell death, as determined by the significant increases in the frequency of early apoptotic cells (Av^+^ 7AAD^-^), late apoptotic cells (Av^+^ 7AAD^+^), and dead cells (Av^-^ 7AAD^+^) after infection. Caspase-3 is activated by the extrinsic (death-ligand *via* caspase-8) as well as intrinsic pathways (mitochondria *via* caspase-9 and caspase-10) and plays a central role in the execution phase of cell apoptosis (24). To confirm the role of apoptosis in CD4 T cell death, we measured active caspase-3 levels in HIV-infected and uninfected cells over time. In line with the Av/7AAD staining, active caspase-3 levels were significantly increased in CD4 T cells with HIV infection (**Figure 1B**). To dissect the behavior of productively infected and bystander cells during HIV infection, we further analyzed active caspase-3 levels in p24^+^ and p24^-^ cells in HIV culture. As shown in **Figure 1C**, active caspase-3 was significantly upregulated in both p24^-^ cells and p24^+^ cells compared to uninfected cells. These results suggest that apoptotic death plays a critical role in CD4 T cell depletion during early HIV infection.

Unlike apoptosis, pyroptosis is a highly inflammatory form of programmed cell death that occurs most frequently upon infection with intracellular pathogens including viruses (25). Pyroptosis requires the function of caspase-1 to promote maturation of IL-1β and IL-18 cytokines, and the immune cells recognize the internal pathogens, release pro-inflammatory cytokines, enlarge, burst, and die. The released cytokines contribute to the damage of surrounding cells – a mechanism involved in CD4 T cell depletion in HIV infection (26, 27). Similar to active caspase-3, active caspase-1 protein was markedly upregulated in HIV-infected cells (**Figure 1D**), in both p24^+^ cells and p24^-^ cells (**Figure 1E**), compared to uninfected cells. These results suggest that HIV infection also drives CD4 T cell pyroptosis.

Ferroptosis is a type of programmed cell death characterized by the loss of glutathione peroxidase 4 (GPX4, a lipid repair enzyme), resulting in unchecked lipid peroxidation and eventual cell death (28). To determine whether ferroptosis plays a role in HIV-mediated cell death, we measured GPX4 expressions in CD4 T cells with or without HIV infection. As expected, GPX4 levels were downregulated in CD4 T cells (**Figures 1F, G** **)**, primarily in p24^-^ cells, with HIV infection (**Figure 1H**). These results indicate increased ferroptosis, particularly in bystander T cells, during HIV infection. Notably, we also measured cell autophagy and found that autophagy markers (p62, LC3B) were also dysregulated, primarily in p24^-^ bystander cells, during HIV infection (data not shown). Taken together, these data indicate that HIV infection promotes CD4 T cell death *via* enhancing cell apoptosis and pyroptosis (especially in p24^+^ cells) and augmenting cell ferroptosis (especially in p24^-^ cells) during early HIV infection.

### The Mechanisms of Survival or Death of Productively Infected and Bystander CD4 T Cells During HIV Infection

**Figure 2:**
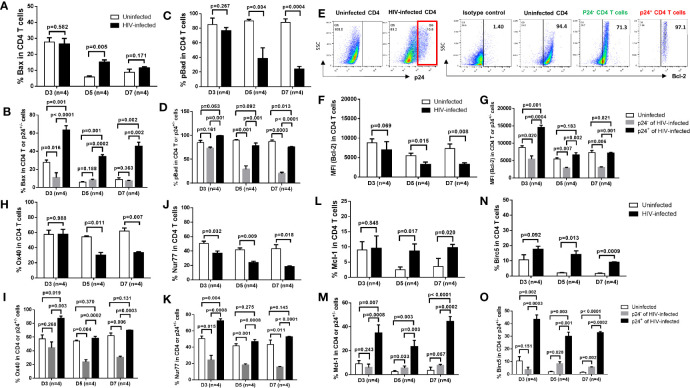
Expression of pro- and anti-apoptotic proteins in productively infected cells and bystander cells during HIV infection. **(A, B)** Summary data of Bax % in HIV-infected (p24^+ -^) and bystander (p24^-^) or uninfected cells at days 3, 5, and 7, as determined by flow cytometry. **(C, D)** Summary data of pBad % in HIV-infected (p24^+^) and bystander (p24^-^) or uninfected cells at days 3, 5, and 7. **(E–O)** Representative dot plots and summary data of Bcl-2%, Ox40% **(H, I)**, Nur77% **(J, K)**, Mcl-1 **(L, M)**, and Birc5% **(N, O)** in HIV-infected (p24^+^ vs. p24^-^) and uninfected cells at days 3, 5, and 7.

To elucidate the mechanisms involved in cell death or survival in productively infected or bystander cells in HIV infection, we examined the expressions of critical pro- and anti-apoptotic or survival proteins in HIV-infected and uninfected cells. As shown in **Figure 2A**, flow cytometry analysis revealed an increase in the expression of pro-apoptotic protein Bax (29) in total CD4 T cells, primarily in p24^+^ cells, in HIV culture compared to uninfected cells (**Figure 2B**). In contrast, we found an overall downregulation of anti-apoptotic proteins such as pBAD, Bcl-2, OX40, and Nur77 (29–32) in total CD4 T cells, primarily in p24^-^ bystander cells rather than p24^+^ cells, in HIV culture compared to uninfected cells (**Figures 2C–K**). Interestingly, while BAD protein is pro-apoptotic, phosphorylated BAD is anti-apoptotic, because canonical anti-apoptotic proteins such as BCL-2/BCL-XL form heterodimers with BAD during BAD-dephosphorylation and this phenomenon triggers BAX/BAK-mediated apoptosis. However, when BAD is phosphorylated by AKT and BCL-2 is released, the free BCL-2 inhibits BAX-triggered apoptosis (30). Moreover, we found that Mcl-1 and BIRC5 (Survivin) - two important survival proteins (32, 33) - were significantly downregulated in TCR-activated, uninfected cells and upregulated in HIV-infected cells, particularly in p24^+^ cells (**Figures 2L–O**). Taken together, these results suggest that p24^+^ and p24^-^ T cells may employ different mechanisms for cell death or survival; i.e., productively infected cells may die or survive *via* upregulation of pro-apoptotic molecules or survival proteins, respectively, whereas bystander cells may die *via* suppression of anti-apoptotic proteins.

### HIV Promotes CD4 T Cell Death by Inducing DNA Damage and Disrupting Telomeres *via* Inhibition of Telomerase During Early Infection

**Figure 3:**
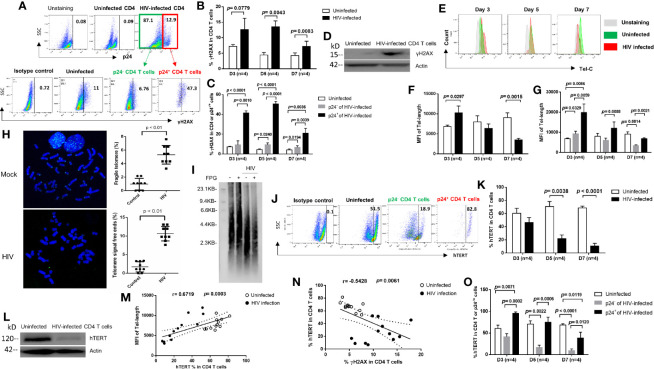
HIV causes CD4 T cell death by inducing DNA damage and disrupting telomeres *via* inhibition of telomerase during early viral infection. **(A)** Representative dot plots and gating strategy of γH2AX levels in p24^+^ and p24^-^ CD4 T cells with HIV infection at day 7. Isotype staining and uninfected CD4 T cells serve as negative controls for flow cytometry. **(B, C)** Summary data of γH2AX % in HIV-infected (p24^+^ vs. p24^-^) and uninfected cells at days 3, 5, and 7. **(D)** Western blot analysis of γH2AX levels in HIV-infected versus uninfected CD4 T cells at day 5. **(E–G)** Representative overlaid histograms and summary data of the mean fluorescence intensity (MFI) of telomere length in HIV-infected (p24^+^ or p24^-^) and uninfected CD4 T cells at days 3, 5, and 7, as determined by Flow-FISH. **(H)** Representative imaging and summary data of meta-FISH analysis of the frequencies (%) of chromatin with fragile telomeres and telomere-free ends in CD4 T cells with or without HIV infection at day 5, measured by confocal microscopy. **(I)** Telomere length in CD4 T cells with or without HIV infection at day 5. Telomeric DNA was treated with or without FPG and analyzed by Southern blot. **(J, K, O)** Representative dot plots and summary data of the human telomerase (hTERT) % in HIV-infected (p24^+^ or p24^-^) and uninfected CD4 T cells at days 3, 5, and 7. **(L)** Western blot analysis of hTERT levels in HIV-infected versus uninfected CD4 T cells at day 5. **(M)** Correlation analysis between hTERT expression and telomere length. **(N)** Correlation analysis between hTERT expression and γH2AX level in HIV-infected and uninfected cells.

Upon cell injury, a dynamic alteration in chromatin structure promotes phosphorylation of the H2A histone family member X (γH2AX) - a marker for DNA double-strand breaks (34, 35). To determine whether HIV induces CD4 T cell death *via* triggering DNA damage, we measured γH2AX during early HIV infection. We found a substantial increase in γH2AX levels in HIV-infected CD4 T cells (**Figures 3A, B** **)**, with higher levels in p24^+^ cells than in p24^-^ cells (**Figure 3C**), compared to uninfected cells. The increases in γH2AX levels were validated by western blot using HIV-infected CD4 T cells after 5 days in culture (**Figure 3D**). These results are in line with the aberrant cell death described above (**Figure 1**).

Since DNA damage causes telomere shortening, and because extremely shortened telomeres lead to cell death (10–17, 36, 37), we hypothesized that the progressive CD4 T cell loss during HIV infection could be, at least in part, due to accelerated telomere erosion. In support of this notion, we have recently reported that CD4 T cells from patients with HIV, HBV or HCV infection are particularly vulnerable to apoptosis due to accumulation of unrepaired telomere erosion (10–17). To determine whether telomere erosion occurs in CD4 T cells during early HIV infection, we measured telomere length by Flow-FISH. As shown in **Figures 3E, F**, HIV-infected CD4 T cells exhibited an initial increase followed by a gradual decrease in telomere length after infection. Counterintuitively, productively infected (p24^+^) CD4 T cells exhibited longer telomeres than p24^-^ bystander cells, which had longer telomeres at initial (day 3) HIV infection compared to uninfected cells, and telomere lengths were shortened in both p24^+^ and p24^-^ cells by day 7 after HIV infection (**Figure 3G**). To confirm these results, we employed the meta-FISH approach to examine telomere status in meta-phase CD4 T cells with or without HIV infection. As shown in **Figure 3H**, the frequencies (%) of cells with fragile telomeres (defined as signals of telomeres on chromosomes that break into segments or stretch due to faulty DNA replication) or telomere-free ends (defined as chromatin ends that do not have detectable telomere signal) were significantly higher in HIV-infected CD4 T cells than uninfected cells at day 5. We also validated these results with Southern blot using CD4 T cells without or with HIV infection at day 5 and observed shortened telomeres in HIV-infected cells compared to the uninfected cells (**Figure 3I**). Notably, we have recently shown that repeated telomeric sequences (TTAGGG) are enriched in guanine (G) - which is particularly sensitive to oxidative stress - are significantly increased in aging T cells during latent HIV infection (15) and form 8-oxoGuanine (8-oxoG) lesions that can be digested by FPG (an 8-oxoG DNA glycosylase). Indeed, Southern blot analysis of treated cellular DNA revealed shorten telomeres in HIV-infected cells (**Figure 3I**). These results indicate that telomeres are significantly shortened with oxidative DNA damage following HIV infection and that 8-oxoG DNA injury may be involved in the process.

Telomeres are elongated by telomerase, which consists of telomerase RNA (TR) and human telomerase reverse transcriptase (hTERT), the catalytic subunit of telomerase for telomere elongation (38). To determine whether the dynamic changes in telomere length in CD4 T cells are due to alterations in the telomerase expression, we measured hTERT levels in CD4 T cells with or without HIV infection using flow cytometry. Consistent with telomere length, TERT was decreased in HIV-infected cells compared to uninfected cells (**Figures 3J, K** **)**. Inhibition of hTERT expression was confirmed by western blot analysis of HIV-infected CD4 T cells (at day 5) (**Figure 3L**). Notably, the percentage of hTERT^+^ cells positively correlated with the median fluorescence intensity (MFI) of telomere length (**Figure 3M**), but negatively correlated with the frequencies of γH2AX^+^ cells within the same cells (**Figure 3N**). Importantly, productively infected (p24^+^) cells expressed greater amounts of telomerase (even more than uninfected controls) compared to p24^-^ bystander cells, although the frequency of hTERT^+^ cells (**Figure 3O**) as well as the MFI of hTERT expression (data not shown) trended downward in both p24^+^ and p24^-^ cells overtime with HIV infection - which is in line with their telomere lengths observed in the same cells. Taken together, these findings suggest that HIV infection accelerates DNA damage and telomere erosion in CD4 T cells and that productively infected cells have longer telomeres than bystander cells due to an increase in telomerase expression.

### TCR Signaling Pathways Are Differentially Regulated in Productively HIV-Infected and Bystander Cells

**Figure 4:**
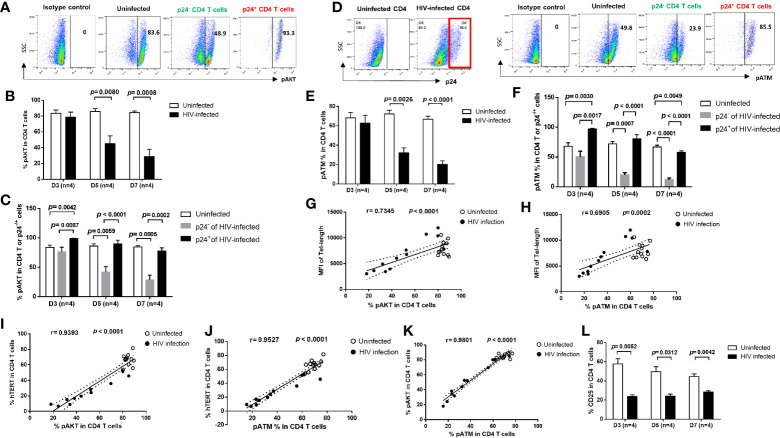
T cell receptor (TCR) signaling pathways are differentially regulated in productively HIV-infected and bystander cells. **(A–C)** Representative dot plots and summary data of the pAKT % in HIV-infected (p24^+^) and bystander (p24^-^) or uninfected D4 T cells at days 3, 5, and 7. **(D–F)** Representative dot blots and summary data of the pATM % in HIV-infected (p24^+^ and bystander (p24^-^) or uninfected CD4 T cells at days 3, 5, and 7, by flow cytometry. **(G, H)** Positive correlation between the MFI of telomere length and the % of pAKT or pATM expressions. **(I–K)** Positive correlation between the % of hTERT, pAKT, and pATM expressions in CD4 T cells with or without HIV infection. **(L)** Percentage of CD25 expression in CD4 T cells with or without HIV infection.

Cells are equipped with DNA damage surveillance and repair machineries to prevent cell death associated with genomic instability (39–41). Accumulation of damaged DNA and apoptotic CD4 T cells indicates an impairment of the TCR signaling and DNA damage repair machinery during viral infection (10–17). We thus hypothesized that progressive CD4 T cell loss during HIV infection could be, at least in part, due to dynamic alterations in the TCR signaling pathways, potentially leading to disruption of telomere integrity and cell longevity. To determine whether these pathways are dysregulated, we analyzed the activation of the PI3K pathways and DNA repair kinases, pAKT and pATM (ataxia-telangiectasia mutated) in HIV-infected and uninfected cells. We found similar expression patterns for these proteins, including pAKT (**Figures 4A–C**) and pATM (**Figures 4D–F**), which were inhibited in total CD4 T cells (**Figures 4B, E** **)**, especially in p24^-^ bystander cells, but were upregulated in p24^+^ cells at the initial phase (day 3) of HIV infection, and then gradually decreased with progressive HIV infection (**Figures 4C, F** **)**. Notably, the percentages of overall pAKT^+^ and pATM^+^ cells positively correlated with the telomere length and telomerase expression levels in total CD4 T cells as well as in p24^+^ and p24^-^ cells during early HIV infection (**Figures 4G–J**). Also, pAKT and pATM exhibited similar expression patterns in HIV-infected and uninfected cells (**Figure 4K**). Concurrently with the inhibition of the TCR signaling pathways, the expression of T cell activation marker CD25 was also inhibited in CD4 T cells with HIV infection (**Figure 4L**). These results indicate that telomere erosion and cell apoptosis are associated with the TCR activation-mediated telomerase and AKT/ATM dynamic changes induced by HIV infection.

### HIV (Gag Protein) Shortens Telomere Length by Diminishing the Telomerase, AKT, and ATM Activities in CD4 T Cells

**Figure 5:**
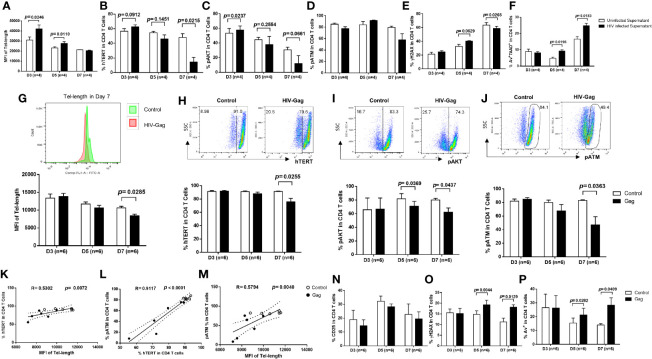
HIV Gag protein shortens telomere length by diminishing telomerase, AKT, and ATM activities in primary CD4 T cells. **(A–F)** Primary CD4 T cells were incubated with supernatants derived from 7-day culture of HIV-infected or uninfected cells in the presence of HIV-entry blockers (Enfuvirtide and Maraviroc) for 3, 5, and 7 days, followed by measuring telomere length, telomerase, pAKT, pATM, γH2AX, and Av/7AAD by flow cytometry. **(G)** Representative overlaid histograms and summary data of the median fluorescence intensity (MFI) of telomere length in Gag-treated and untreated CD4 T cells at days 3, 5, and 7, measured by Flow-FISH. **(H–J)** Representative dot plots and summary % of the hTERT, pAKT, and pATM expressions in Gag-treated and untreated CD4 T cells at days 3, 5, and 7. **(K–M)** Positive correlation between the MFI of telomere length and the % of hTERT, pATM, and hTERT, as well as pATM expression and telomere length in CD4 T cells with or without Gag treatment. **(N–P)** Summary data of the % of CD25^+^, γH2AX^+^, and Av^+^ cells in CD4 T cells treated with or without HIV Gag.

How productively infected cells affect bystander cells during HIV infection remains unknown. One possibility is that HIV particles or proteins are released from infected cells into the culture supernatants and inhibit those uninfected, bystander cells. To test this possibility, we incubated uninfected CD4 T cells with culture supernatants of HIV-infected (at day 7) or uninfected cells in the presence of HIV-entry blockers for 3, 5, and 7 days, followed by measuring telomere length, telomerase, AKT, and ATM activities. Notably, without HIV entry blockers, we observed similar changes (as shown above in **Figures 3**, **4**) in telomere length, telomerase, AKT, and ATM (data not shown). We then included the HIV-entry blocker T-20 (Enfuvirtide), a small peptide that binds to the HIV gp41 heptad repeat and inhibits the structural changes necessary for the virus to fuse with CD4^+^ T cell - and Maraviroc (Selzentry), an entry inhibitor that specifically blocks chemokine receptor CCR5 that HIV uses as a co-receptor to bind and enter human helper T cells - in the culture to exclude HIV entry and infection of cells. Interestingly, CD4 T cells treated with HIV-supernatants plus entry blockers, which inhibit active infection but retain the live virus in the culture media, exhibited a prolonged telomere length at day 3 and day 5 similar to early HIV-infected cells at day 3 (shown in **Figures 3D, E** **)** compared to control cultures. However, blocking HIV entry and infection reversed telomere length inhibition by active HIV infection at days 5-7 (compare **Figure 5A** with **Figures 3D, E** **)**. Correspondingly, blocking HIV active infection of the cells partially reversed the virus-mediated inhibition of hTERT, pAKT, and pATM (compare **Figures 5B–D** with **Figure 3J** and **Figures 4B, E**, respectively). Also, HIV infection-induced DNA damage and cell apoptosis were partially reversed by the viral entry blockers, as evidenced by the levels of γH2AX (**Figure 5E**) and AV/7AAD staining (**Figure 5F**) compared to HIV-infected cells without viral blockers (**Figures 3B** and **1A**).

Since HIV appears to induce telomere erosion through inhibition of telomerase, AKT, and ATM activities, we next sought to determine which specific HIV protein can drive such an effect on CD4 T cells. To this end, we incubated primary CD4 T cells with major recombinant HIV proteins (gp120, p24, Tat, p7, Nef, and Gag) for 3, 5, and 7 days, followed by measuring telomere length, hTERT, pAKT, and pATM by flow cytometry. Intriguingly, among the HIV proteins examined, only HIV Gag could significantly shorten telomere length, only at day 7 after treatment (**Figure 5G**). Correspondingly, the expressions of hTERT, pAKT, and pATM were inhibited in primary CD4 T cells treated with HIV Gag for 7 days (**Figures 5H–J**). Moreover, the MFI of telomere length positively correlated with the frequencies of hTERT^+^ cells treated with or without HIV Gag protein for 7 days (**Figure 5K**), and both the frequency of hTERT^+^ cells and MFI of telomere length closely correlated with the frequency of pATM^+^ cells in CD4 T cells exposed to HIV Gag protein for 7 days (**Figures 5L, M** **)**. Importantly, HIV Gag treatment inhibited T cell activation, enhanced DNA damage, and promoted cellular apoptosis, as demonstrated by the decreased expression of early T cell activation marker CD25 (**Figure 5N**) and increased staining of γH2AX (**Figure 5O**) and Av (**Figure 5P**) in treated CD4 T cells over time. However, the other HIV proteins, such as gp120, p24, Tat, p7, and Nef, did not elicit such effects on telomere length and its related regulatory proteins in multiple experiments (data not shown). Taken together, these results indicate that HIV (Gag protein) plays a role in the shortening of telomeres, likely through inhibition of the hTERT and AKT/ATM signaling pathways.

### Inhibition of the ATM Pathway Promotes Survival of HIV-Infected Cells and Rescues AKT/Telomerase Activities *via* Inhibiting HIV Infection and DDR

**Figure 6:**
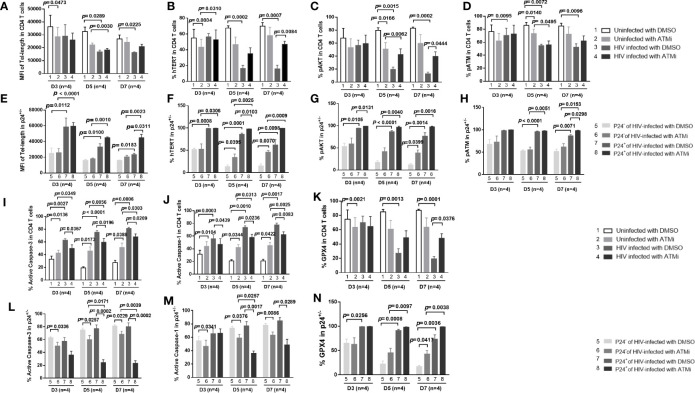
ATM blockade promotes HIV-infected cell survival *via* rescuing AKT and telomerase activities by inhibiting HIV infection and DNA damage response (DDR). **(A–D)** HIV-infected and uninfected CD4 T cells were treated with DMSO control or ATM inhibitor for 3, 5, and 7 days, followed by measuring telomere length, hTERT, pAKT, and pATM by flow cytometry. **(E–H)** Summary data of the MFI of telomere length, the % of hTERT, pAKT, and pATM expressions in p24^+^ and p24^-^ cells following HIV infection with ATMi or DMSO treatment for 3, 5, and 7 days. **(I–K)** Summary data of the % of active caspase-3, active caspase-1, and GPX4 expressions in HIV-infected and uninfected CD4 T cells treated with DMSO or ATMi for 3, 5, and 7 days. **(L–N)** Summary data of the % of active caspase-3, active caspase-1, and GPX4 expressions in p24^+^ and p24^-^ cells following HIV infection with ATMi or DMSO treatment for 3, 5, and 7 days.

We have previously shown that ATM is not only a marker for DDR (10–17), but also a marker for T cell activation, as demonstrated by the close correlation between pATM and pAKT and hTERT expression patterns during T cell activation and HIV infection (**Figure 4**). Therefore, we hypothesized that inhibition of the ATM pathway would suppress T cell activation, HIV infection, and DDR. To test this hypothesis, we activated CD4 T cells by anti-CD3/CD28 stimulation, followed by HIV infection in the presence of DMSO or ATM inhibitor (ATMi, 10 μM KU60019) for 3, 5, and 7 days, and then measured the telomere length, and hTERT, pAKT, and pATM expressions. As we reported previously (14, 15), healthy CD4 T cells treated with ATMi showed inhibition of telomere length, and correspondingly, the expression levels of hTERT, pAKT and pATM were inhibited in uninfected cells by ATMi compared to those treated by DMSO (**Figures 6A–D**, 2 vs. 1). Similar to the results described above (**Figures 3**, **4**), HIV infection promoted telomere erosion, along with inhibition of hTERT, pAKT, and pATM expressions (**Figures 6A–D**, 3 vs. 1). However, ATMi treatment resulted in a partial recovery of telomere length and a partial restoration of hTERT, pAKT, and pATM expressions, especially at day 5-7 of HIV infection with ATMi vs. DMSO treatment (**Figures 6A–D**, 4 vs. 3).

**Figure 7:**
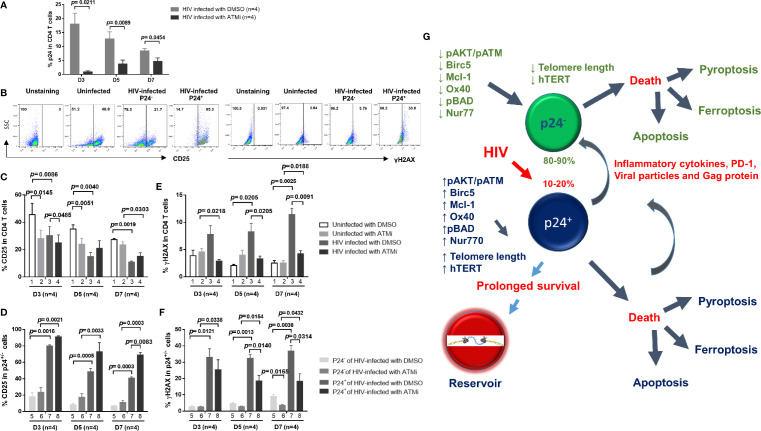
Blockade of ATM promotes HIV-infected cell survival *via* inhibiting T cell activation, HIV infection, and DNA damage. **(A)** p24 expression in HIV-infected CD4 T cells treated with DMSO or ATM inhibitor (ATMi). **(B–F)** Summary data of the % of CD25 and γH2AX expressions in uninfected and HIV-infected (p24^+^ and p24^-^) CD4 T cells treated with DMSO or ATMi for 3, 5, and 7 days. **(G)** A schematic model of the fate of productively infected CD4 T cells and bystander cells during early HIV infection. HIV promotes CD4 T cell death through enhancing various programmed cell death pathways, including apoptosis and pyroptosis (especially in p24^+^ cells) or ferroptosis (especially in p24^-^ cells) during early viral infection. Mechanistically, HIV infection promotes secretion of inflammatory cytokines as well as viral particles and proteins (such as Gag) that can differentially regulate the pro- and anti-apoptotic proteins, such as Bad, pBad, Bcl-2, OX40, Nur77, Mcl-1, and Birc5, in p24^+^ and p24^-^ cells. HIV infection also dysregulates T cell receptor (TCR) signaling pathways, in particular PI3K (such as AKT/ATM) and telomerase hTERT activities, and thus affects telomere length and cell survival or death machineries in p24^+^ and p24^-^ cells. While productively infected (p24^+^) cells experience more death than p24^-^ bystander cells, p24^+^ cells appear to exhibit survival activity, thus favoring HIV reservoir formation and latency establishment. Specifically, productively infected CD4 T cells show prolonged telomeres, increased levels of telomerase, and more activated TCR signaling pathways compared to bystander CD4 T cells, indicating the involvement of cell survival and pro-reservoir machineries during HIV infection. Importantly, blocking the ATM pathway promotes uninfected cell death but enhances virus-infected cell survival, and blocking HIV infection enhances overall cell survival, both through regulating the signaling pathways involved in the maintenance of genomic telomere integrity. These results indicate that HIV-infected and uninfected cells employ different mechanisms for survival and death during HIV infection.

We further analyzed these expression patterns in productively infected cells and bystander cells in HIV culture. Again, as we observed a survival potential in productively infected cells (**Figures 3**, **4**), we found that p24^+^ cells exhibited longer telomeres and higher levels of hTERT, pAKT, and pATM expressions than p24^-^ cells in HIV cultures with DMSO treatment (**Figures 6E–H**, 7 vs. 5). We also observed the same patterns in p24^+^ cells with the ATMi treatment (**Figures 6E–H**, 8 vs. 6). However, compared to HIV-infected, DMSO-treated cultures, ATMi treatment resulted in a partial restoration of telomere length as well as hTERT, pAKT, and pATM expressions in both p24^-^ cells and p24^+^ cells, especially at day 5-7 of HIV infection with ATMi vs. DMSO treatment (**Figures 6E–H**, 6 vs. 5 and, 8 vs. 7).

Likewise, we asked whether ATM inhibition could promote uninfected CD4 T cell death and rescue HIV-infected cells. To this end, cultured CD4 T cells with TCR activation were infected with HIV in the presence of DMSO or ATM inhibitor for 3, 5, and 7 days, followed by measuring active caspase-3, active caspase-1, and GPX4 expressions for cell apoptosis, pyroptosis, and ferroptosis, respectively. In line with their telomere length and hTERT/pAKT/pATM levels (**Figures 6A–D**), healthy CD4 T cells treated with ATMi exhibited an increase in the active caspase-3 and active caspase-1 expression levels, but a decrease in the GPx4 expression compared to those treated with DMSO (**Figures 6I–K**, 2 vs. 1). Similar to our observations shown in **Figure 1**, HIV infection promoted CD4 T cell apoptosis, pyroptosis, and ferroptosis, as evidenced by the increase in the levels of active caspase-3 and active caspase-1, and the decrease in GPx4 expression (**Figures 6I–K**, 3 vs. 1). However, ATMi treatment resulted in a partial recovery of these effectors, especially at day 5-7 after HIV infection and ATMi treatment (**Figures 6I–K**, 4 vs. 3). Also, we analyzed programmed cell death in productively infected cells and bystander cells in HIV culture. Again, as we observed a survival potential in productively infected cells (**Figure 1**), and noticed that p24^+^ cells exhibited similar or lower levels of cell apoptosis, pyroptosis, and ferroptosis than p24^-^ cells in HIV cultures with DMSO treatment (**Figures 6L–N**, 7 vs. 5) and with the ATMi treatment (**Figures 6L–N**, 8 vs. 6). However, compared with DMSO treatment of HIV-infected cultures, ATMi treatment resulted in a partial rescue of cell death in both p24^-^ cells and p24^+^ cells, especially at day 5–7 of HIV infection with ATMi vs. DMSO treatment (**Figures 6L–N**, 6 vs. 5 and, 8 vs. 7).

We have previously shown that ATM inhibition induces telomeric DNA damage, PI3K/AKT inhibition, and apoptotic death in healthy CD4 T cells (14, 15). Why ATM inhibition elicits an opposite effect in HIV-infected CD4 T cells remains unknown. Since HIV infection in the setting of TCR stimulation induces DNA damage and cell apoptosis (14, 15), and since ATM activation serves as a marker for early DDR (10–12), we further hypothesized that ATM inhibition could inhibit T cell activation, and thus HIV infection as well as subsequent DDR. To test this hypothesis, cultured CD4 T cells with TCR activation were infected with HIV in the presence of DMSO or ATM inhibitor (ATMi, 10 μM KU60019) for 3, 5, and 7 days, followed by measuring the levels of T cell early activation marker CD25, HIV infection marker p24, and DNA damage marker γH2AX. Indeed, HIV infection was inhibited by ATMi treatment, as evidenced by the significant decrease p24^+^ cell frequency (**Figure 7A**). This repressed HIV infection is likely related to ATMi-mediated inhibition of CD4 T cell activation that is a prerequisite of HIV infection, and the representative graphs of CD25 and γH2AX was shown in (**Figure 7B**), as shown by the overall decrease in CD25 expression in HIV-inhibited cells (**Figure 7C**, 3 vs. 1). ATMi also inhibited T cell activation in uninfected cells (**Figure 7C**, 2 vs. 1). However, ATMi treatment partially restored CD25 expression in HIV-infected cells at days 5-7 (**Figure 7C**, 4 vs. 3) in both p24^-^ and p24^+^ cells (**Figure 7D**, 6 vs. 5, and 8 vs. 7). Correspondingly, γH2AX expression was increased by HIV infection (**Figure 7E**, 3 vs. 1), and ATMi treatment increased γH2AX levels in uninfected cells (**Figure 7E**, 2 vs. 1), but decreased it in HIV-infected cells (**Figure 7E**, 4 vs. 3) in both p24^-^ and p24^+^ cells (**Figure 7F**, 6 vs. 5, and 8 vs. 7). Taken together, these results suggest that inhibition of the ATM pathway promotes the death of healthy CD4 T cells but protects survival of HIV-infected cells by regulating T cell activation, DDR, and HIV infection.

## Discussion

The mechanisms involved in the death and survival of productively infected CD4 T cells and bystander cells after initial HIV infection are crucial for the depletion of CD4 T cells and/or development of a replication-competent virus reservoir. Here we focused on CD4 T cell death/survival dynamics following initial HIV infection by infecting primary CD4 T cells with a wild-type HIV-1 strain and assaying the cell death/survival mechanisms, including telomeric dynamics and TCR signaling pathways. Our findings suggest that HIV infection, in general, promotes CD4 T cell programmed cell deaths, including apoptosis, pyroptosis, and ferroptosis, but productively infected cells behave differently compared to bystander cells, likely due to activation of specific cellular survival/death programs during early viral infection. Specifically, our data suggest that productively infected cells exhibit greater telomere lengths and express higher levels of hTERT, pAKT, and pATM upon initial infection, indicating a possible pro-survival, pro-reservoir mechanism at play during early HIV infection. Importantly, blocking virus-entry into these cells attenuated HIV-induced telomerase, pAKT, and pATM inhibition as well as telomere erosion and cell death. Additionally, ATM inhibition promoted survival of HIV-infected (especially p24^+^) cells *via* rescuing telomere length and telomerase and AKT activities by inhibiting T cell activation, HIV infection, and DDR, thus reflecting CD4 T cell survival and death machineries during HIV infection.

Telomeres are repeating hexameric sequences of DNA at chromosome ends associated with a complex of shelterin proteins. Telomere integrity is a key feature of linear chromosomes that preserves genome stability and function, whereas telomere attrition is a hallmark of cellular senescence that drives cell dysfunction or apoptosis (36, 37). Telomerase is essential for maintaining the replicative capacity of T cells by preserving shortening of telomere length and thereby cell senescence (38, 39). The hTERT is the catalytic subunit of telomerase and its expression positively correlates with telomerase activity in human cells (38, 39). Notably, hTERT nuclear translocation and activity depend upon AKT activation (40, 41), which is closely associated with ATM activation, as we observed in this current study (**Figure 4**). Therefore, blocking the ATM pathway will lead to inhibition of AKT and T cell activation, and subsequent HIV infection and DDR. This is in line with previous reports showing that hTERT can protect cells from apoptosis, in addition to protecting them from senescence, and hTERT has also been used to immortalize cells for long-term survival (42, 43).

HIV induces telomerase activity in macrophages, conferring resistance to oxidative stress and possibly safeguarding one of its known reservoirs (44). Our data suggest that early HIV infection in CD4 T cells is also characterized by increased telomerase activity, AKT/ATM activities, and telomere lengths in p24^+^ cells compared to p24^-^ cells, although all these activities eventually trend down along with HIV infection compared to uninfected cells. While cell death can still progress *via* any of the multiple programmed cell death pathways (apoptosis, ferroptosis, and pyroptosis), overall these p24^+^ cells appear to maintain telomeric integrity (**Figure 3F**) more robustly than p24^-^ bystander cells at the early stage of HIV infection. Mechanistically, this integrity appears to be supported by the AKT/ATM pathways, which regulate telomerase activity and telomere dynamics, which are upregulated in p24^+^ productively infected cells (**Figures 3**, **4**). Counterintuitively, blocking the ATM pathway abrogates the HIV-induced inhibition of telomerase activity and telomere length, particularly in the p24^+^ cells. This increased pAKT/pATM activity in p24^+^ cells is not evident in p24^-^ cells, and is likely a response to the viral infection itself rather than to exogenous exposure of inflammatory cytokines, soluble antigens, or inhibitory molecules within the cellular milieu.

Could the preservation of telomeres upon initial infection potentially favor HIV reservoir formation? Dynamically, infected CD4 T cells undergo continual activation and integration of HIV genetic components within the host cell genome. Only those cells that survive cell death can go on to establish viral reservoirs. Thus, preserving telomeric integrity has the potential to facilitate survival of the infected cells and thus favors the establishment of HIV reservoirs. Similar to our finding, Reynoso et al (44). reported induction of telomerase activity in monocyte-derived macrophages following HIV-1 infection, and these macrophages exhibited less DNA damage after oxidative stress compared to the uninfected cells. This effect on telomerase activity correlated with p24 production, and accordingly the authors proposed that HIV “hitchhikes” on telomerase activity in macrophages to use the cells as a reservoir. Our data support this proposal in that this pro-reservoir type of function may also occur in the CD4 T cell population, but our study also suggests an initial increase in telomere length in infected CD4 T cells that was not observed in macrophage. A recent study using a latency model of HIV infection has also suggested that HIV peptide-induced, reactivated p24^+^ CD4 T cells express higher levels of hTERT and pAKT (45). However, our studies were carried out in a more through, calculated, kinetic and rigid way to demonstrate this notion, again highlighting a potential survival mechanism for productively infected CD4 T cells to drive HIV reservoir formation.

As noted above, activation of AKT and ATM signaling differed significantly in productively infected cells compared to bystander cells and correlated quite well with both hTERT and telomere lengths. The PI3K/Akt pathway is known to be a key pathway for cell survival (46–48). Specifically, tuning of the AKT pathway by HIV Nef and its blockade by protease inhibitors has been shown to limit HIV-1 recovery from a latently infected T cell line (49). Our data suggest that persistent inhibition of the AKT and ATM pathways by HIV Gag results in decreased telomerase activity and telomere length that favors cell death, whereas the activation of the PI3K/AKT and ATM survival pathways early on during HIV infection may maintain telomeric integrity in a milieu that otherwise clearly favors cell survival. Based on these data, we propose a model for early HIV infection (**Figure 7G**) that supports the differential effects observed in p24^+^ vs. p24^-^ CD4 T cells. According to this model, a significant death in both cell populations occurs *via* multiple death programs, including apoptosis, necroptosis, and pyroptosis. We demonstrate that p24^+^ CD4 T cells exhibit protective survival signaling, in particular, *via* the pAKT/pATM pathways that promote maintenance of telomere length compared to bystander T cells. However, there is clear evidence of telomere shortening and impaired AKT/ATM pathways in bystander CD4 T cells, perhaps driven by viral antigens such as HIV Gag and/or the inflammatory cytokine milieu in the culture system. Indeed, we have revealed, by two independent cytokine array assays, that HIV-infected CD4 T cells can secrete significantly higher levels of multiple pro-inflammatory cytokines into cultures over time, including IL-1a, IL-18, TNFB, fractalkine, and GCSF, but release lower levels of other mediators such as IL-10 and sCD40L. We have also discovered considerable PD-1 expressions on the surface of HIV-infected cells compared to uninfected cells (data not shown), suggesting both soluble factors and direct cell-cell contact mediated inhibitory mechanisms are possibly involved in the effects of productively infected cells on bystander cells. This paradigm warrants further studies.

Our study focused on primary CD4 T cells rather than latent cell lines, and thus has limitations inherent to examining early HIV infection. Cell infectivity was based upon the presence of p24 antigen positivity, and it is possible that at least some cells were at the very early stage of productive infection and did not produce sufficient p24. Also, due to limitations in p24 antibody’s background and sensitivity, the number of infected cells could be over- or under-estimated. However, as a whole, some delineation must occur to separate populations and this measure appears reasonable. We also did not assess the extent of HIV reservoir formation over time in our primary cell cultures, focusing only on the initial events after HIV infection. This was in large part due to the fact, as our data show, that HIV-infected primary CD4 T cells suffered very dramatic and significant cell death in the cultural milieu and that generation of the reservoir cells is the exception rather than the rule. The main strength of this study is the use of primary cells and infectious HIV-1 virus to delineate the differences and death/survival mechanisms between productively HIV-1-infected cells and bystander cells instead of cell lines and partial HIV-1 constructs; one limitation of this study, however, is that there is no bridge to *in vivo* data, such as *ex vivo* analysis of virus-infected and uninfected cells from HIV patients. This is simply because we could not detect p24 antigens in CD4 T cells derived from HIV-infected patients presenting with specific viral loads (HIV RNA levels) due to the interruption of ART.

In summary, our study uncovered that HIV infection induces rapid and dramatic cell death in both productively infected and bystander cells, and this cell death is associated with loss of telomeric integrity *via* dysregulation of the telomerase and PI3K pathways. However, survival signaling by the AKT, ATM, and hTERT pathways are activated in productively infected CD4 T cells and appears to drive the maintenance of telomere integrity. Future studies will focus on delineating how this survival signaling mechanism promotes the formation of viral reservoirs, on revealing events that can prevent the establishment of latent infection, and on interrupting the virus cycle to eliminate reservoirs and cure HIV.

## Data Availability Statement

The raw data supporting the conclusions of this article will be made available by the authors, without undue reservation.

## Ethics Statement

The studies involving human participants were reviewed and approved by ETSU/VA IRB committee.

## Author Contributions

DC performed most of the experiments, SK, LW, LNN, XD, LNTN, BKCT, JZ, MS, ZKL and ZYL participated in some experiments. XYW and ZDM provided technical support. ME, JYZ, SN, and JPM offered intellectual input for troubleshooting and discussion of the findings. ZQY supervised the project and wrote the manuscript, with the help of all other authors. All authors contributed to the article and approved the submitted version.

## Funding

This work was supported by National Institutes of Health grants R01AI114748 and R21AI138598, and S10OD021572; VA Merit Review Awards 1I01BX002670 and 1I01BX004281; and DoD Award PR170067 (to ZQY). This publication is the result of work supported with resources and the use of facilities at the James H. Quillen Veterans Affairs Medical Center. The contents in this publication do not represent the views of the Department of Veterans Affairs or the United States Government.

## Conflict of Interest

The authors declare that the research was conducted in the absence of any commercial or financial relationships that could be construed as a potential conflict of interest.

## References

[1] Okoye AA Picker LJ. CD4(+) T-cell depletion in HIV infection: mechanisms of immunological failure. Immunol Rev (2013) 254:54–64. doi: 10.1111/imr.12066 23772614 PMC3729334

[2] Vidya Vijayan KK Karthigeyan KP Tripathi SP Hanna LE. Pathophysiology of CD4+ T-Cell Depletion in HIV-1 and HIV-2 Infections. Front Immunol (2017) 8:580. doi: 10.3389/fimmu.2017.00580 28588579 PMC5440548

[3] Piconi S Trabattoni D Gori A Parisotto S Magni C Meraviglia P. Immune activation, apoptosis, and Treg activity are associated with persistently reduced CD4^+^ T-cell counts during antiretroviral therapy. AIDS (2010) 24:1991–2000. doi: 10.1097/QAD.0b013e32833c93ce 20651586

[4] Lederman MM Calabrese L Funderburg NT Clagett B Medvik K Bonilla H. Immunologic failure despite suppressive antiretroviral therapy is related to activation and turnover of memory CD4 cells. J Infect Dis (2011) 204:1217–26. doi: 10.1093/infdis/jir507 PMC3218674 21917895

[5] Tyagi M Bukrinsky M. Human immunodeficiency virus (HIV) latency: the major hurdle in HIV eradication. Mol Med (2012) 18:1096–108. doi: 10.2119/molmed.2012.00194 PMC3475336 22692576

[6] Eisele E Siliciano RF. Redefining the viral reservoirs that prevent HIV-1 eradication. Immunity (2012) 37:377–88. doi: 10.1016/j.immuni.2012.08.010 PMC3963158 22999944

[7] Chomont N El-Far M Ancuta P Trautmann L Procopio FA Yassine-Diab B. HIV reservoir size and persistence are driven by T cell survival and homeostatic proliferation. Nat Med (2009) 15:893–900. doi: 10.1038/nm.1972 19543283 PMC2859814

[8] Chavez L Calvanese V Verdin E. HIV latency is established directly and early in both resting and activated primary CD4 T cells. PloS Pathog (2015) 11:e1004955. doi: 10.1371/journal.ppat.1004955 26067822 PMC4466167

[9] Imamichi H Crandall KA Natarajan V Jiang MK Dewar RL Berg S. Human immunodeficiency virus type 1 quasispecies that rebound after discontinuation of highly active antiretroviral therapy are similar to the viral quasispecies present before initiation of therapy. J Infect Dis (2001) 183:36–50. doi: 10.1086/317641 11106537

[10] Ji Y Dang X Nguyen LNT Nguyen LN Zhao J Cao D. Topological DNA damage, telomere attrition and T cell senescence during chronic viral infections. Immun Ageing (2019) 16:12. doi: 10.1186/s12979-019-0153-z 31285747 PMC6591813

[11] Dang X Ogbu SC Zhao J Nguyen LNT Cao D Nguyen LN. Inhibition of topoisomerase IIA (Top2α) induces telomeric DNA damage and T cell dysfunction during chronic viral infection. Cell Death Dis (2020) 11:196. doi: 10.1038/s41419-020-2395-2 32193368 PMC7081277

[12] Cao D Zhao J Nguyan LN Nguyen LNT Khanal S Dang X. Disruption of Telomere Integrity and DNA Repair Machineries by KML001 Induces T Cell Senescence, Apoptosis, and Cellular Dysfunctions. Front Immunol (2019) 10:1152. doi: 10.3389/fimmu.2019.01152 31191531 PMC6540964

[13] Nguyen L Zhao J Cao D Dang X Wang L Lian J. Inhibition of TRF2 accelerates telomere attrition and DNA damage in naïve CD4 T cells during HCV infection. Cell Death Dis (2018) 9:900. doi: 10.1038/s41419-018-0897-y 30185784 PMC6125360

[14] Zhao J Dang X Zhang P Nguyen LN Cao D Wang L. Insufficiency of DNA repair enzyme ATM promotes naïve CD4 T cell loss in chronic hepatitis C virus infection. Cell Discov (2018) 4:16. doi: 10.1038/s41421-018-0015-4 29644094 PMC5891503

[15] Zhao J Nguyen LNT Nguyen LN Dang X Cao D Khanal S. ATM Deficiency Accelerates DNA Damage, Telomere Erosion, and Premature T Cell Aging in HIV-Infected Individuals on Antiretroviral Therapy. Front Immunol (2019) 10:2531. doi: 10.3389/fimmu.2019.02531 31781094 PMC6856652

[16] Schank M Zhao J Wang L Li Z Cao D Nguyen LN. Telomeric injury by KML001 in human T cellsinduces mitochondrial dysfunction through thep53-PGC-1α pathway. Cell Death Dis (2020) 11:1030. doi: 10.1038/s41419-020-03238-7 33268822 PMC7710715

[17] Khanal S Tang Q Cao D Zhao J Nguyen LN Oyedeji OS. Telomere and ATM dynamics in CD4 T cell depletion in active and virus-suppressed HIV infection. J Virol (2020) 94:e01061–20. doi: 10.1128/JVI.01061-20 PMC7592222 32907975

[18] Shete A Suryawanshi P Godbole S Pawar J Paranjape R Thakar M. HIV-infected CD4+ T cells use T-bet-dependent pathway for production of IL-10 upon antigen recognition. Scand J Immunol (2016) 83:288–96. doi: 10.1111/sji.12422 27028319

[19] Jones RB Ndhlovu LC Barbour JD Sheth PM Jha AR Long BR. Tim-3 expression defines a novel population of dysfunctional T cells with highly elevated frequencies in progressive HIV-1 infection. J Exp Med (2008) 205:2763–79. doi: 10.1084/jem.20081398 PMC2585847 19001139

[20] Vali B Jones RB Sakhdari A Sheth PM Clayton K Yue FY. HCV-specific T cells in HCV/HIV co-infection show elevated frequencies of dual Tim-3/PD-1 expression that correlate with liver disease progression. Eur J Immunol (2010) 40:2493–505. doi: 10.1002/eji.201040340 20623550

[21] Martins LJ Bonczkowski P Spivak AM De Spiegelaere W Novis CL DePaula-Silva AB. Modeling HIV-1 Latency in Primary T Cells Using a Replication-Competent Virus. AIDS Res Hum Retroviruses 2016:32:187–193. doi: 10.1089/aid.2015.0106 PMC4761834 26171776

[22] Kimura M Stone RC Hunt SC Skurnick J Lu X Cao X. Measurement of telomere length by the Southern blot analysis of terminal restriction fragment lengths. Nat Protoc (2010) 5:1596–607. doi: 10.1038/nprot.2010.124 21085125

[23] Adachi A Gendelman HE Koenig S Folks T Willey R Rabson A. Production of acquired immunodeficiency syndrome-associated retrovirus in human and nonhuman cells transfected with an infectious molecular clone. J Virol (1986) 59:284–91. doi: 10.1128/JVI.59.2.284-291.1986 PMC253077 3016298

[24] Boatright KM Salvesen GS. Mechanisms of caspase activation. Curr Opin Cell Biol (2003) 15:725–31. doi: 10.1016/j.ceb.2003.10.009 14644197

[25] Fink SL Cookson BT. Apoptosis, Pyroptosis, and Necrosis: Mechanistic Description of Dead and Dying Eukaryotic Cells. Infect Immun (2005) 73:1907–16. doi: 10.1128/IAI.73.4.1907-1916.2005 PMC1087413 15784530

[26] Doitsh G Galloway NLK Geng X Yang Z Monroe KM Zepeda O. Cell death by pyroptosis drives CD4 T-cell depletion in HIV-1 infection. Nature (2014) 505:509–14. doi: 10.1038/nature12940 PMC4047036 24356306

[27] Doitsh G Greene WC. Dissecting How CD4 T Cells Are Lost During HIV Infection. Cell Host Microbe (2016) 19:280–91. doi: 10.1016/j.chom.2016.02.012 PMC4835240 26962940

[28] Yang WS Stockwell BR. Ferroptosis: Death by Lipid Peroxidation. Trends Cell Biol (2016) 26:165–76. doi: 10.1016/j.tcb.2015.10.014 PMC4764384 26653790

[29] Adefolaju GA Theron KE Hosie MJ. Effects of HIV protease, nucleoside/non-nucleoside reverse transcriptase inhibitors on Bax, Bcl-2 and apoptosis in two cervical cell lines. BioMed Pharmacother (2014) 68:241–51. doi: 10.1016/j.biopha.2013.08.007 24011602

[30] Scheid MP Schubert KM Duronio V. Regulation of bad phosphorylation and association with Bcl-x(L) by the MAPK/Erk kinase. J Biol Chem (1999) 274:31108–13. doi: 10.1074/jbc.274.43.31108 10521512

[31] Harant H Lindley IJ. Negative cross-talk between the human orphan nuclear receptor Nur77/NAK-1/TR3 and nuclear factor-kappaB. Nucleic Acids Res (2004) 32:5280–90. doi: 10.1093/nar/gkh856 PMC521667 15466594

[32] Kuo HH Ahmad R Lee GQ Gao C Chen HR Ouyang Z. Anti-apoptotic Protein BIRC5 Maintains Survival of HIV-1-Infected CD4^+^ T Cells. Immunity (2018) 48:1183–94. doi: 10.1016/j.immuni.2018.04.004 PMC6013384 29802019

[33] Warren CFA Wong-Brown MW Bowden NA. BCL-2 family isoforms in apoptosis and cancer. Cell Death Dis (2019) 10(3):177. doi: 10.1038/s41419-019-1407-6 30792387 PMC6384907

[34] Furuta T Takemura H Liao ZY Aune GJ Redon C Sedelnikova OA. Phosphorylation of histone H2AX and activation of Mre11, Rad50, and Nbs1 in response to replication-dependent DNA double-strand breaks induced by mammalian DNA topoisomerase I cleavage complexes. J Biol Chem (2003) 278:20303–12. doi: 10.1074/jbc.M300198200 12660252

[35] Kuo LJ Yang LX. Gamma-H2AX - a novel biomarker for DNA double-strand breaks. In Vivo (2008) 22:305–9. 18610740

[36] Arkus N. A mathematical model of cellular apoptosis and senescence through the dynamics of telomere loss. J Theor Biol (2005) 235:13–32. doi: 10.1016/j.jtbi.2004.12.016 15833310

[37] Blackburn EH. Telomere states and cell fates. Nature (2000) 408:53–6. doi: 10.1038/35040500 11081503

[38] Blackburn EH. Telomeres and telomerase: their mechanisms of action and the effects of altering their functions. FEBS Lett (2005) 579:859–62. doi: 10.1016/j.febslet.2004.11.036 15680963

[39] Shay JW Wright WE. Telomeres and telomerase: three decades of progress. Nat Rev Genet (2019) 20:299–309. doi: 10.1038/s41576-019-0099-1 30760854

[40] Chung J Khadka P Chung IK. Nuclear import of hTERT requires a bipartite nuclear localization signal and Akt-mediated phosphorylation. J Cell Sci (2012) 125:2684–97. doi: 10.1242/jcs.099267 22366458

[41] Plunkett FJ Franzese O Finney HM Fletcher JM Belaramani LL Salmon M. The loss of telomerase activity in highly differentiated CD8+CD28-CD27- T cells is associated with decreased Akt (Ser473) phosphorylation. J Immunol (2007) 178:7710–9. doi: 10.4049/jimmunol.178.12.7710 17548608

[42] Hathcock KS Chiang YJ Hodes RJ. In vivo regulation of telomerase activity and telomere length. Immunol Rev (2005) 205:104–13. doi: 10.1111/j.0105-2896.2005.00267.x 15882348

[43] Luiten RM Pène J Yssel H Spits H. Ectopic hTERT expression extends the life span of human CD4+ helper and regulatory T-cell clones and confers resistence to oxidative stress-induced apoptosis. Blood (2003) 101:4512–9. doi: 10.1182/blood-2002-07-2018 12586632

[44] Reynoso R Wieser M Ojeda D Bönisch M Kühnel H Bolcic F. HIV-1 induces telomerase activity in monocyte-derived macrophages, possibly safeguarding one of its reservoirs. J Virol (2012) 86:10327–37. doi: 10.1128/JVI.01495-12 PMC3457250 22787205

[45] Suryawanshi P Godbole S Pawar J Thakar M Shete A. Higher expression of human telomerase reverse transcriptase in productively-infected CD4 cells possibly indicates a mechanism for persistence of the virus in HIV infection. Microbiol Immunol (2018) 62:317–26. doi: 10.1111/1348-0421.12585 29577368

[46] Bartek J Lukas J. DNA damage checkpoints: from initiation to recovery or adaptation. Curr Opin Cell Biol (2007) 19:238–45. doi: 10.1016/j.ceb.2007.02.009 17303408

[47] Dupre A Boyer-Chatenet L Gautier J. Two-step activation of ATM by DNA and the Mre11-Rad50-Nbs1 complex. Nat Struct Mol Biol (2006) 13:451–7. doi: 10.1038/nsmb1090 16622404

[48] Awasthi P Foiani M Kumar A. ATM and ATR signaling at a glance. J Cell Sci (2015) 128:4255–62. doi: 10.1242/jcs.169730 26567218

[49] Kumar A Abbas W Colin L Khan KA Bouchat S Varin A. Tuning of AKT-pathway by Nef and its blockade by protease inhibitors results in limited recovery in latently HIV infected T-cell line. Sci Rep (2016) 6:24090. doi: 10.1038/srep24090 27076174 PMC4831010

