# Corrigendum: A matter of life or death: Productively infected and bystander CD4 T Cells in early HIV infection

**DOI:** 10.3389/fimmu.2022.937057

**Published:** 2022-07-26

**Authors:** Dechao Cao, Sushant Khanal, Ling Wang, Zhengke Li, Juan Zhao, Lam Nhat Nguyen, Lam Ngoc Thao Nguyen, Xindi Dang, Madison Schank, Bal Krishna Chand Thakuri, Jinyu Zhang, Zeyuan Lu, Xiao Y. Wu, Zheng D. Morrison, Mohamed El Gazzar, Shunbin Ning, Jonathan P. Moorman, Zhi Q. Yao

**Affiliations:** ^1^ Center of Excellence for Inflammation, Infectious Disease and Immunity, James H. Quillen College of Medicine, East Tennessee State University, Johnson City, TN, United States; ^2^ Division of Infectious, Inflammatory and Immunologic Diseases, Department of Internal Medicine, Quillen College of Medicine, East Tennessee State University, Johnson City, TN, United States; ^3^ Hepatitis (HCV/HBV/HIV) Program, James H. Quillen VA Medical Center, Department of Veterans Affairs, Johnson City, TN, United States

**Keywords:** AKT, ATM, HIV, telomerase, telomere, T cell death, survival

In the original article, there was a mistake in **Figure 1D** as published. The representative flow dot plots were misplaced. The corrected **Figure1D** appears below.

**Figure 1:**
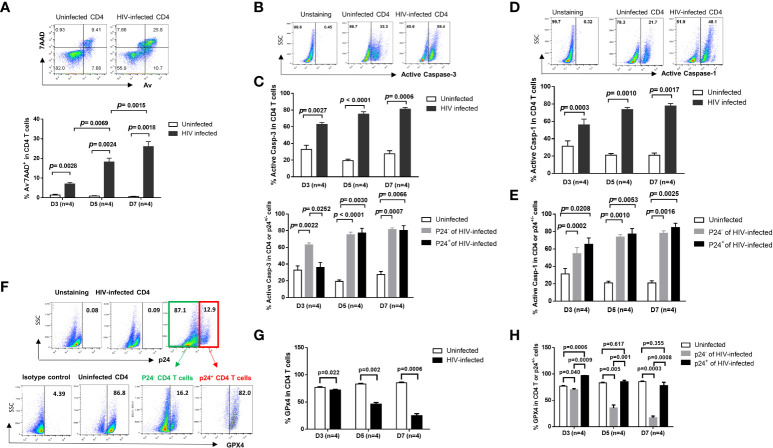
HIV induces CD4 T cell death in productively infected and bystander cells during early infection via different mechanisms. **(A)** Representative dot plots (day 7) and summary of the percentage (%) of Av and 7AAD levels in HIV-infected and uninfected CD4 T cells, as determined by flow cytometry. **(B, C)** Representative dot plots and summary data showing active caspase-3% in productively HIV-infected (p24+) and bystander (p24-) or uninfected cells at days 3, 5, and 7 in cultures. **(D, E)** Representative dot plots and summary data of active caspase-1% in HIV-infected (p24+) and bystander (p24-) or uninfected cells at day 3, day 5, and day 7. **(F–H)** Representative dot plots and summary data of GPX4% in HIV-infected (p24+) and bystander (p24-) or uninfected cells at days 3, 5, and 7.

The authors apologize for this error and state that this does not change the scientific conclusions of the article in any way. The original article has been updated.

## Publisher’s Note

All claims expressed in this article are solely those of the authors and do not necessarily represent those of their affiliated organizations, or those of the publisher, the editors and the reviewers. Any product that may be evaluated in this article, or claim that may be made by its manufacturer, is not guaranteed or endorsed by the publisher.

